# High frequency of ocular toxoplasmosis in Quindío, Colombia and risk factors related to the infection

**DOI:** 10.1016/j.heliyon.2021.e06659

**Published:** 2021-04-05

**Authors:** Jorge Enrique Gómez-Marín, Juliana Muñoz-Ortiz, Manuela Mejía-Oquendo, José Y. Arteaga-Rivera, Nicolás Rivera-Valdivia, María Cristina Bohórquez-Granados, Stefany Velasco-Velásquez, Gabriela Castaño-de-la-Torre, John Alejandro Acosta-Dávila, Laura Lorena García-López, Elizabeth Torres-Morales, Mónica Vargas, Juan David Valencia, Daniel Celis-Giraldo, Alejandra de-la-Torre

**Affiliations:** a Grupo GEPAMOL, Facultad de Ciencias de la Salud, Universidad del Quindío, Armenia, Carrera 15 #12N, Colombia; b Grupo de Investigación Escuela Barraquer, Escuela Superior de Oftalmología del Instituto Barraquer de América, Bogotá, Avenida Calle 100 No. 18A – 51, Colombia; c Facultad de Medicina, Pontificia Universidad Javeriana, Bogotá, Carrera. 7 # 40 – 62, Colombia; d Grupo de Investigación en Neurociencias NeURos, Escuela de Medicina y Ciencias de la Salud, Universidad Del Rosario, Bogotá, Carrera 24 #63C-69, Colombia

**Keywords:** Ocular toxoplasmosis, *Toxoplasma gondii*, Screening, Risk factors, Retinochoroiditis, Colombia

## Abstract

**Objectives:**

To determine the frequency of retinochoroidal lesions by ocular toxoplasmosis and their relationships with risk factors, in residents of two districts with high exposure to *Toxoplasma,* in Armenia-Quindío, Colombia.

**Methods:**

Cross-sectional analyses of fundoscopy screening, serological tests, and questionnaires were performed to determine risk factors associated with ocular toxoplasmosis retinochoroidal lesions. Differences in proportions were analyzed using the chi-squared test.

**Results:**

Of 161 individuals examined, 17 (10.5%) exhibited retinochoroidal scars suggestive of old inactive *Toxoplasma gondii* infection. All 17 individuals were seropositive for *T. gondii* antibodies. Consumption of bottled water was protective against *T. gondii* infection among individuals in this study. There were no specific epidemiological risk factors associated with ocular toxoplasmosis retinochoroidal lesions.

**Conclusion:**

Ocular toxoplasmosis is an important cause of visual impairment in Armenia-Quindío, Colombia. The consumption of boiled or bottled water is a major preventive public health measure to reduce infection by *T. gondii* and the subsequent onset of OT.

## 1 Introduction

Ocular toxoplasmosis (OT) is an infection caused by *Toxoplasma gondii,* an obligate intracellular protozoan that belongs to the phylum Apicomplexa [1, 2]. *T. gondii* exists in nature in three forms: the oocyst, tissue cyst, and tachyzoite [1]. The oocyst contains infective sporozoites that are shed to the environment by felids, the definitive host [1]. Furthermore, the tissue cyst contains bradyzoites, which are found in intermediate and definitive hosts. Finally, the tachyzoite is a rapidly-dividing proliferative form of the parasite that is present during the acute phase of infection [1].

Sources of infection for humans include consumption of unboiled water, poorly washed vegetables or fruits, direct contact with soil containing oocysts, or ingestion of tissue cysts in undercooked meat [3]. Notably, these sources of infection vary among geographical regions and depend on the presence of *T. gondii* in the environment, the populations of wild and domestic felids, and cultural and hygienic habits [4, 5].

*T. gondii* exhibits tropism for the eye and can cause OT, which most frequently affects retinochoroidal tissue in immunocompetent individuals [2]. Although *T. gondii* infection is less prevalent in immunocompetent patients, the resulting disease exhibits considerable severity and can cause extensive damage to the central nervous system [2]. OT is one of the most common forms of infection caused by *T. gondii* in humans. *T. gondii* is the main etiological agent of posterior uveitis in immunocompetent patients; it can cause visual impairment and is the second most common cause of congenital blindness [2].

The prevalence of OT differs among geographical regions worldwide; it is most prevalent in South America, Central America, the Caribbean, and parts of tropical Africa [6, 7]. The annual incidence of OT in Colombia is approximately three per 100,000 individuals [8].

The diagnosis of OT is made mainly by clinical observation of a focal necrotizing retinochoroidal lesion, which appears as a whitish-yellow region with a blurred margin, with or without an accompanying old lesion [2]. Serological tests (e.g., serum anti-*Toxoplasma* titers of IgM and IgG) or the use of DNA amplification by PCR for direct detection of *T. gondii* may aid in the diagnosis [9, 10]. Macular scars cause moderate to severe disability, involving altered visual field in 65.2% of patients with OT, diminished visual acuity in 27% of patients with OT [11], and legal blindness in at least one eye in 24% of patients with OT [12].

Few studies have examined epidemiological risk factors linked to OT [13, 14, 15]. Risk factors for OT are generally presumed to be like those for non-ocular toxoplasmosis; however, some studies in Brazil revealed that cohabitation with cats or dogs and the consumption of raw or undercooked meat were risk factors for toxoplasmosis infection in general but not particularly for OT [16]. Furthermore, in Colombia, it was reported that ROP16 virulent alleles of *T. gondii* were found to be linked to ocular toxoplasmosis and, differently, ROP16 non-virulent alleles were detected in meat samples, which may suggest that the main source for ocular *T. gondii* infection is not undercooked meat [17].

This study aimed to identify the proportion of people with OT retinochoroidal lesions in two urban districts with frequent exposure to *Toxoplasma* and compare the risk factors for toxoplasmosis in people with and without ocular lesions in the city of Armenia-Quindío, Colombia, where a high prevalence of OT has been previously described [8, 18].

## 2 Methods

### 2.1 Study population

This cross-sectional study included inhabitants of two districts located at the center and south of the city (districts 1: “Guaduales de la Villa” and 2: “La Universal”) in Armenia-Quindío. In districts 1 and 2, the total populations were 461 and 577, respectively. These districts were selected because they were previously found to have a high prevalence of *Toxoplasma* DNA in the stools of their home feline pets being 10% in district 1 and 36% in district 2 [19]. This was not a study about the prevalence of OT in the city of Armenia, but was focused on examining the extent of the infection in these two districts and how visual health was affected by *Toxoplasma* infection; as well as what were the risk factors related to this frequency. All residents of each district were invited to undergo visual screening during community meetings, church and home visits. All included individuals provided written informed consent to participate in the study, following a full explanation of the study protocol. Children were allowed to participate in the study if the consent was obtained from a parent or legal guardian. Individuals were excluded if they could not undergo a complete ophthalmological examination.

### 2.2 Ophthalmological examination

Visual health screening was performed by two ophthalmology residents of the *Escuela Superior de Oftalmología - Instituto Barraquer de América*, then confirmed by a retinologist and a uveologist. Visual acuity was measured using a Snellen chart at a distance of 6 m; external examination and biomicroscopy were performed with a portable slit lamp. A drop of tropicamide 1% with phenylephrine 5% was applied in participants who did not exhibit a narrow-angle in the anterior chamber. Subsequently, the posterior segment of the eye was examined using indirect ophthalmoscopy under pupillary dilation to determine the presence of active retinochoroiditis or retinochoroidal scars. The clinical diagnosis of OT was made based on previously described criteria [20, 21].

### 2.3 Serological tests

All participants were sampled for serological analysis. 10 ml of the peripheral blood sample was obtained and centrifuged 1,500 g × 10 min on the day of collection; the resulting serum supernatant was stored at −20 °C until the test could be performed one or two weeks later. Serum samples were assayed using indirect ELISA (Enzyme-linked Immunosorbent Assay) for *T. gondii* IgG (Human, Wiesbaden, Germany), using an Stat Fax 3200 ELISA microplate reader spectrophotometer (Awareness Technology Inc., Palm City, Florida, USA). The tests were done according to the kit's manufacturer instructions, considering a sample as negative if it presented <10 UI/ml. For this assay, the reported sensitivity is 96.1% and specificity is 99.2% [22]. IgM anti-*Toxoplasma* was analyzed only in people presenting retinal lesions by using the VIDAS® TOXO enzyme-linked fluorescent immunoassay (ELFA) from Biomerieux (France). This provides an automated qualitative detection of anti-*Toxoplasma gondii* IgM antibodies in human serum. For IgM, the level <0.55 UI/ml was considered as negative and >0.65 UI/ml was considered as positive.

### 2.4 Questionnaire

Participants were interviewed by trained medical students. Questionnaires included general demography data, including age and medical history, as well as epidemiological and sociodemographic characteristics. In Colombia, the socioeconomic level of the population is determined using the SISBEN classification, an established socioeconomic index, where the lowest level corresponds to SISBEN 0 and the highest to SISBEN 6. Risk factors assessed in the questionnaire included the main source of water in the household—“open water source” was defined as any open water body including lakes, springs, rivers, streams, ponds, swamps, and dams— as well as the consumption of raw meat or not properly washed vegetables, and other possible sources of infection. Specific questions were: Do you drink bottled or boiled water? Do you drink un-boiled water occasionally? Do you drink beverages prepared with un-boiled water? Do you drink water from an open water source? Do you drink water directly from the faucet? Do you eat undercooked meat frequently or occasionally? Do you eat at restaurants frequently or occasionally -at least once a week-? Do you own cats at home? Do you have contact with cats under 6 months of age? Do you eat unwashed vegetables?

### 2.5 Statistical analysis

A participant database was established and validated in Microsoft Excel (Microsoft Corp., Redmond, WA, USA). Results are expressed as median [min–max] for continuous variables and N (%) for categorical variables. Differences in proportions were analyzed using the chi-squared test or Fisher's exact test, as appropriate. One-way analysis of variance was used to evaluate differences in the mean levels of anti-*Toxoplasma* IgG antibodies among study participants. Odds ratios and 95% confidence intervals were calculated. SPSS version 25 (SPSS Inc. IBM, Chicago, USA) statistical program was used to perform analysis. P values <0.05 were considered statistically significant.

### 2.6 Ethics approval

The present study was performed following the ethical principles for research involving human beings established by the Declaration of Helsinki, the Belmont Report, and Colombian Resolution 008430 of 1993. The confidentiality of participants' information was preserved based on the Habeas data law (Statutory law 1581 of 2012). This study was approved by the Ethics Committee of Universidad Del Quindío (Act 19 of 7 June 2019).

### 2.7 Consent for publication

All patients who agreed to participate signed informed consent. In minors b assent and informed consent were obtained from the participants and parents, respectively.

## 3 Results

### 3.1 Sociodemographic status

One hundred sixty-one individuals (322 eyes) were examined during the screening, 68% were women, and the median age was 50 years. All participants were of Hispanic ethnicity. Most participants were of SISBEN socioeconomic level 2 (49.1%) and 76.4% of participants did not achieve higher education. More detailed information about demographic characteristics is found in Table 1.

**Table 1:** Sociodemographic status of the 161 participants in the visual screening.

Variable	Groups	n (N = 161)	%
Age (years)∗	1–26	42	26.1
	27–59	67	41.6
	≥60	52	32.3
Sex	Female	110	68.3
	Male	51	31.7
SISBEN∗∗	1	32	19.9
	2	79	49.1
	3	49	30.4
	4	1	0.6
Educational level	No studies	8	5.0
	Elementary	64	39.8
	High school	55	34.2
	Technician	7	4.3
	University (higher education)	27	16.7
District	Guaduales de la Villa (Total population = 461)	68	42.2
	La Universal (Total population = 577)	93	57.8

∗ The range between ages was of 1–91 years, with a median of 50 years.

∗∗ SISBEN: for its initials in Spanish (Identification System of Potential Beneficiaries of Social Programs), is a Colombian socio-economic index.

### 3.2 Sociodemographic status differences between districts

There were not statistically significant differences in the percent of participants between district 1 (14.7%) and district 2 (16.1%) regarding the total number of habitants of each district (p = 0.64). Participants mean age was higher in district 2 than in district 1 (54 versus 43; p = 0.023). There were not significant differences in the percent of people that belonged to SISBEN lower than 3 (76.5% in district 1 and 63.4% in district 2; p = 0.11). No significant differences in gender distribution were observed (p = 0.12) nor in the level of education (p = 0.95) between districts. Complete data of the population is presented in Supplementary File 1.

### 3.3 Clinical and serological findings

**Figure 1:**
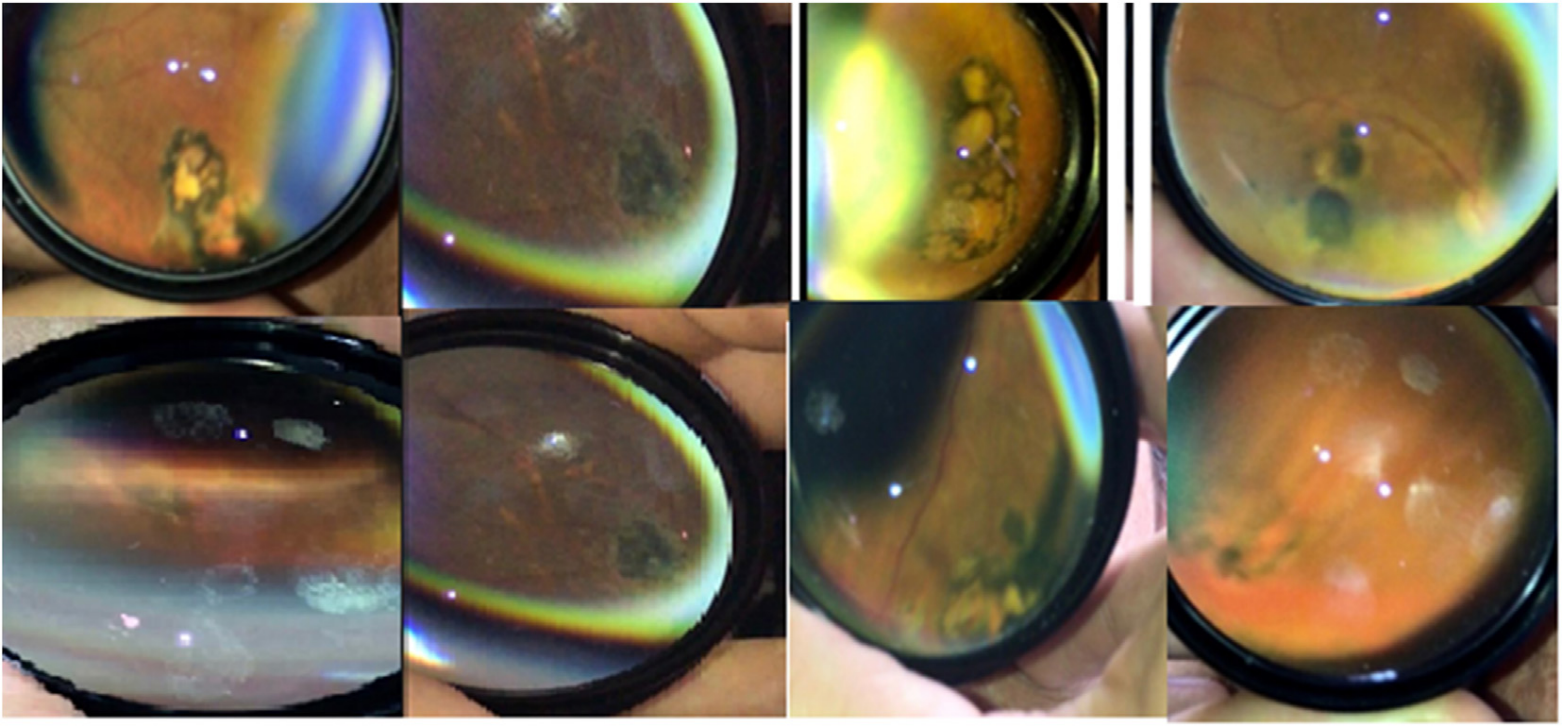
Images of eight typical retinochoroidal scars found during indirect ophthalmoscopy in the eye fundus screening program in two districts in Armenia (Colombia) in 2019.

Clinical examination revealed that 17 (10.5%) participants had retinochoroidal scars; none exhibited signs of active lesions. The most common symptom in 12/17 participants was blurred vision. One participant exhibited bilateral nystagmus associated with congenital toxoplasmosis. The congenital origin of the infection was established because typical signs were present at birth. Other important ophthalmological findings were optic disc pallor and eye convergence insufficiency**.** IgG antibodies against *T. gondii* were detected in 112/161 (69.5%) participants. All participants with retinochoroidal scars were positive for anti-*T. gondii*-specific IgG antibodies but not corresponding IgM antibodies; this indicated that all infections were chronic. Relevant clinical and serological findings in participants with retinochoroidal lesions are summarized in Table 2. Representative lesions are shown in Figure 1.

**Table 2:** Clinical and serological findings in participants with retinochoroidal lesions.

Patient No.	Age (years)	Gender	Affected Eye	Visual Acuity	Number of Lesions	Mean size of Lesions (optic disk diameter)	Size of the biggest lesion (optic disk diameter)	Localization	Toxoplasma IgG	Toxoplasma IgM
1	26	F	OS	20/80	1	0,25	0,25	P	+	-
2	17	F	OS	20/50	2	1,56	3	P	+	-
3	56	F	ORS	20/20	2	0,12	0,12	P	+	-
4	41	F	OD	20/20	2	1,75	2	P	+	-
5	84	F	OD	20/100	2	1,75	2,5	P	+	-
6	52	M	OD	20/100	1	0,12	0,12	C/P	+	-
7	59	M	ORS	20/160	5	1,5	5	C/P	+	-
8	50	F	OS	20/25	1	1	1	P	+	-
9	33	F	OD	20/25	1	1,5	1,5	P	+	-
10	37	F	OS	20/20	1	0,25	0,25	P	+	-
11	77	M	OD	CF1M	1	8	8	C	+	-
12	79	F	ORS	CF1M	3	6,3	8	C/P	+	-
13	24	M	OS	20/70	1	0,25	0,25	C	+	-
14	70	F	OS	20/20	1	0,25	0,25	P	+	-
15	61	F	OD	20/40	1	1	1	P	+	-
16	87	M	OS	20/20	1	0,25	0,25	C	+	-
17	71	F	OS	20/30	2	0,81	1,5	P	+	-

F = female; M = male; OD = right eye; OS = left eye; CF = counting fingers; C = central (macular); P = peripheral; Ig = immunoglobulin.

### 3.4 Risk factors analysis

Analysis of potential risk factors indicated that the consumption of bottled water was protective against *T. gondii* infection based on the comparison between *T. gondii* IgG-negative and IgG-positive participants (Table 3). When comparing potential risk factors between *T. gondii* infected participants with and without ocular lesions, no statistically significant differences were found (Table 4).

**Table 3:** Risk factors associated with anti-*T. gondii* IgG positive results in exposed vs. non-exposed.

Risk factor	Anti-*T. gondii* IgG positive results in exposed vs. non-exposed n/N (%)	OR	IC 95%	p value
Drinking only bottled or boiled water	23/42 (54.7%) vs. 89/119 (74.7%)	0.40	0.19–0.85	**0.015∗**
Cats at home	34/50 (68%) vs. 78/111 (70.2%)	0.84	0.43–1.84	0.772
Contact with cats ages less than 6 months-old	8/10 (80%) vs. 104/151 (68.8%)	1.8	0.37–8.84	0.459
Undercooked meat	14/26 (53.8%) vs. 98/135 (72.6%)	0.44	0.18–1.04	0.057
Commune (Guaduales vs. Universal)	51/68 (75%) vs. 61/93 (65.5%)	0.63	0.31–1.27	0.200

∗Two-tailed statistically significant correlation (p < 0.050).

**Table 4:** Analysis for risk factors associated with ocular lesions in people positive for anti-*T. gondii* IgG.

Risk factor	Ocular lesions in exposed vs. non-exposed n/N (%)	OR	IC 95%	p value
Drinking bottled water	5/23 (21.7%) vs. 12/89 (13.4%)	1.78	0.55–5.70	0.325
Contact with cats	5/34 (14.7%) vs. 12/78 (15.3%)	0.94	0.30–2.93	0.927
Contact with cats ages less than 6 months-old	1/8 (12.5%) vs. 16/104 (15.3%)	0.78	0.09–6.82	0.827
Undercooked meat	2/14 (14.2%) vs. 15/98 (15.3%)	0.92	0.18–4.54	0.921
Commune (Guaduales vs. Universal)	7/61 (11.4%) vs. 10/51 (19.6%)	0.53	0.18–1.51	0.232

## 4 Discussion

OT is an important visual health problem in Colombia. A high prevalence of retinochoroidal scars (6%) was previously observed in a population of young university students in Colombia [18]. Notably, 10%–20% of *T. gondii* infections in immune-competent adults and children are symptomatic [23]. Ocular symptoms in acquired OT include blurred vision, scotoma, ocular pain, photophobia, epiphora, or loss of central vision; such symptoms may be easily distinguishable when they first occur [8, 24]. Blurred vision and floaters were reported by individuals with OT in our study; however, retinochoroidal scars were incidental findings in most participants (88%). Studies conducted in Europe reported that 16%–76.5% of patients with OT exhibited strabismus due to macular lesions, 5.4%–59% exhibited unilateral microphthalmia, 3%–53% exhibited cataracts, 1.5%–11.8% exhibited retinal detachment, 1.5%–76.5% exhibited optic nerve atrophy, 1.5% exhibited iridocyclitis, 0.8%–28% exhibited nystagmus, and 0.8%–5.3% exhibited glaucoma [12, 25, 26]. In our study, only one participant exhibited bilateral nystagmus associated with low vision in both eyes; this individual was also the only one diagnosed with congenital disease. The high prevalence of OT in Colombia and its associated complications, based on our present and prior studies [18, 27] indicate the need for urgent public health measures to facilitate early detection of OT through mandatory ophthalmological screening in sites with a high prevalence of the infection.

Visual impairment in patients with OT was evaluated in a study that included 154 consecutive patients with active retinochoroidal lesions: 24% developed blindness in one eye, while 1% developed blindness in both eyes [12]. Legal blindness was caused predominantly by macular lesions (80% of affected eyes), followed by retinal detachment (13% of affected eyes) and optic nerve atrophy (7% of affected eyes) [12]. In the present study, two individuals diagnosed with OT exhibited legal blindness; both of them exhibited a macular scar, while one exhibited optic nerve atrophy.

The seroprevalence of toxoplasmosis varies worldwide. The overall age-adjusted seroprevalence in a study conducted in the United States was 22.5% (95% confidence interval: 21.1%–23.9%) [28]; it was 7.84% in China [29], 20.3% in India [30], and 34.47% in Iran [31]. In our study, the seroprevalence of toxoplasmosis was 69.5%, which is consistent with the results of a previous study done in Colombia [32].

In a European multicenter study performed in the year 2000, 30%–63% of infections were attributed to the consumption of undercooked or cured meat products, while 6%–17% was attributed to soil contact [33]. Previous studies in Brazil found no particular risk factors related to OT [13, 16, 34]. These findings suggest that host genetic susceptibility is related to the development of the disease. The TA and AA alleles in the IFNγ promoter (SNP rs24305619) were present in 44% and 51% of patients with OT, respectively, whereas the TT allele was present only in 4.3% of these patients; notably, the TT allele was frequent (40%) in patients who had non-ocular *T. gondii* infection [35]. Also, parasite factors can play a role; in one previous investigation we found a noticeable difference in ROP16 virulent factor allele distribution, that was more frequent in samples obtained from OT patients [17].

Present findings confirm that water can be the most important source of *T. gondii* infections in our study region [36]. Chlorine treatment does not eliminate *Toxoplasma* oocysts from drinkable water, and we demonstrated a high frequency of *Toxoplasma* DNA in tap water from the same region of the present study [37]. Currently, we are applying a monitoring program for protozoa in drinkable water and we expect to obtain data about the reduction of infection from new measures such as the ultraviolet treatment of water [38].

## 5 Limitations

One of the limitations of this study is that we used a convenience non-probabilistic sample size, which resulted in a small sample with a low statistical power. This may have prevented the identification of statistically significant potential risk factors. This can be observed in the fact that we found the consumption of undercooked meat very close of being a protective factor, which is a totally unexpected result. Nevertheless, finding a statistically significative protective factor (bottled water consumption) even with a small sample size, indicates the strength of this particular protective factor.

Another limitation is that the risk factor assessment was based on patient's interrogation. Therefore, a memory bias may have been present. However, this bias was controlled by using a standardized questionnaire and resolving all the doubts that arose while filling the questionnaire.

## 6 Conclusions

In conclusion, we found a high rate of ocular lesions due to toxoplasmosis through eye fundoscopy screening of habitants of two districts in Armenia-Quindío, Colombia. We found that, in this region, it is important to promote the consumption of boiled or bottled water as a major preventive public health measure to reduce infection by *T. gondii* and the subsequent onset of OT. Our study supports mandatory eye fund screening as necessary for regions with high prevalence of toxoplasmosis.

## Declarations

### Author contribution statement

Jorge Enrique Gómez-Marín and Alejandra de-la-Torre: Conceived and designed the experiments; Performed the experiments; Analyzed and interpreted the data; Contributed reagents, materials, analysis tools or data; Wrote the paper.

Manuela Mejía-Oquendo, Daniel Celis-Giraldo and Juliana Muñoz-Ortiz: Conceived and designed the experiments; Performed the experiments; Analyzed and interpreted the data; Wrote the paper.

Stefany Velasco-Velásquez: Performed the experiments; Analyzed and interpreted the data; Wrote the paper.

José Y. Arteaga-Rivera, Nicolás Rivera-Valdivia, María Cristina Bohórquez-Granados, Gabriela Castaño de-la-Torre, John Alejandro Acosta-Dávila, Laura Lorena García-López, Elizabeth Torres-Morales, Mónica Vargas and Juan David Valencia: Performed the experiments; Wrote the paper.

### Funding statement

M. Mejía-Oquendo, D. Celis-Giraldo, J. Valencia and L. García-López were supported by Ministerio de Ciencia, Tecnología e Innovación (Colombia) (Grant ID:773-2018).

### Data availability statement

Data will be made available on request.

### Declaration of interests statement

The authors declare no conflict of interest.

### Additional information

No additional information is available for this paper.

## Appendix A Supplementary data

Dataset.xls Dataset.xls
